# Improving the mental health of farmers: what types of remote support are acceptable, feasible, and improve outcomes? A feasibility RCT

**DOI:** 10.1007/s44192-023-00054-1

**Published:** 2024-01-04

**Authors:** Kate Lamont, Hugo C. van Woerden, Emma King, Charlotte Wendelboe-Nelson, Roger W. Humphry, Cameron Stark, Chris Williams, Margaret Maxwell

**Affiliations:** 1 Scotland’s Rural College (SRUC), Scotland, UK; 2 University of the Highlands and Islands, Scotland, UK; 3 University of Stirling, Scotland, UK; 4 Glasgow University, Scotland, UK

## Abstract

**Background:**

The farming community have high rates of poor mental health, and are relatively ‘hard to reach’ with mental health services. The aim of this study was therefore to undertake a feasibility RCT, based on two mental health interventions. These were (1) CBT based ‘Living Life to the Full for Farming Communities’ (LLTTF-F; www.llttf.com), and (2) a holistic social and emotional support service delivered by the Royal Scottish Agricultural Benevolent Institution (RSABI). The feasibility was supplemented by process evaluation.

**Methods:**

This feasibility study aimed to recruit 40 individuals from the farming community who were experiencing a common health problem defined as a score of >  = 8 on PHQ-9. A snowball approach was used to recruit interested individuals who had an association with farming. An initial telephone call screened for eligibility and obtained consent to randomisation to the two specified interventions, or to a thirdly group receiving a combination of both LLTTF-F and ‘Social and emotional support’. Participants were permitted to override the randomised option if they expressed a strong preference before the interventions began.

**Results:**

Thirty-two participants provided baseline and three-month data. All three interventions showed positive improvements on PHQ-9 scores as follows: the ‘combined intervention’ mean baseline score was 18.1 compared to 12.0 at 3-month follow-up (mean change 6.1). ‘Social and emotional support’ mean baseline score was 11.3 compared to 6.7 at 3-month follow-up (mean change 4.6). ‘LLTTF-F CBT-based intervention only’ mean baseline score was 11.8 compared to 4.5 at 3-month follow-up (mean change 7.3). The retention rate was 81% at three months.

In a sub-group of the LLTTF-F CBT-based intervention online materials were supplemented by telephone guided support. This approach received very positive feedback.

**Conclusions:**

Recruitment from the farming community required intense effort, and good engagement can then be retained for at least three months. There is evidence that the interventions used were feasible, and tentative evidence that they had a demonstrable effect on mental wellbeing, with the LLTTFF providing the largest effect on PHQ-9 scores.

*Trial Registration Number* ISRCTN27173711, submitted 25/08/2023, confirmed 22/092023.

**Supplementary Information:**

The online version contains supplementary material available at 10.1007/s44192-023-00054-1.

## Introduction

Recent systematic reviews indicate that farmers are at particular risk of mental health disorders [1, 2]. Depression in the farming community is increasing and suicide rates are among the highest in any occupational group in the UK [3]. Depression in farmers increases with age, and is higher in males, and symptoms of depression are associated with a 13% increase in sex-adjusted and age-adjusted mortality [4–7]. Farmers are also more likely to report thinking that ‘life is not worth living’. It is currently estimated that one agricultural worker per week takes their own life in the UK [3]. Risk of suicide is also higher amongst those working in specific agricultural roles such as harvesting crops and rearing animals (almost twice the national UK average) [3].

Throughout this study, we refer to farmers as including all farm workers and those who are part of the wider agricultural community, including unpaid workers and family members. This includes those who are part time farmers who simultaneously have some paid employment.

Social factors and community characteristics have an important mediating role in the mental health of the farming population [8, 9]. Farmers are more likely to turn to their own communities for support than to health or social work authorities, with many preferring to engage with advice from respected members within their communities, such as veterinary surgeons (Vets), or use anonymous sources of support such as the internet or self-help booklets [10, 11].

CBT based interventions have a strong evidence base for the management of common mental health problems [12] and are often well accepted in rural settings [13, 14]. CBT has the advantage that it can be completed in a relatively short period of time compared with other talking therapies. Its structured nature means it can be provided in different formats (including remote and self-directed formats), and it teaches practical strategies that can be used in everyday life. These qualities may appeal to farmers.

Psychological and social interventions provide different types of support, and combined approaches may be optimal [15, 16]. Our two candidate interventions therefore included the CBT based ‘Living Life to the Full for Farming Communities’ (LLTTF-F) and the Royal Scottish Agricultural Benevolent Institution’s (RSABI) social and emotional support service (see below). These interventions were delivered separately or in combination providing three arms to our RCT.

### Assessing feasibility

Before undertaking a full-sized RCT, the preferences of farmers for interventions, feasibility, and uptake, need to be established. This project therefore sought to establish a ‘best-candidate’ intervention to incorporate in a larger RCT by: identifying a preferred candidate intervention for addressing mental health problems in farmers; assessing the potential to recruit farmers; and testing a potential study outcome measure (PHQ-9).

The study approach was informed by focus groups with farmers, Vets, and other farm advisors. The qualitative aspects of this study have been published elsewhere [17].

The holistic social and emotional support service (including home visits and/or telephone support) was provided by RSABI. The self-help CBT based intervention (LLTTF-F), was based on previous research [18, 19]. LLTTF-F can be delivered on-line or in booklet format, and can be provided with or without telephone guided support. The ‘counselling’ aspect of the social support intervention, was primarily a ‘listening ear’, whereas the LLTTF-F CBT, was a structured and theoretically (cognitive behavioural therapy) based approach with support to engage with the intervention through problem solving techniques.

## Methods

A mixed-methods approach was used to assess the preferences of farmers. This was considered important to establish feasible parameters to include in larger ‘best-candidate’ RCT.

### Theoretical framework

The study’s theoretical framework aligns with ‘pragmatism’ as a research philosophy, where the research question is the important determinant of methods. This combines both positivist and interpretivist positions within the scope of a single study, depending on the nature of the research questions [20, 21]. The study also adopted an approach that was consistent with the sentiments of ‘participatory action research’, with a focus was on transforming the lives of socially marginalised populations. Participatory action research was particularly used to engage an advisory/reference group using appreciative enquiry [22]. More detail on the advisory group and qualitative aspects of the study are provided elsewhere [17].

### Sample size

A sample size calculation, was conducted on the basis of an unpaired t-test, with the required number of observations in each arm of the study, based on an effect size from a published paper reporting a Cohen’s difference, d, of 0.66 on the EQ-VAS scale [23]. A statistical power of 80%, and a statistical significance threshold of 5% was assessed within the statistical software R, based on the pwr.t.test () function within the package “pwr” [24]. The sample size calculation recommended 42 observations in each arm of the hypothetical study considered. However, given the practical logistics of a feasibility study, a decision was made to recruit a smaller sample of 40 individuals spread across all three arms of the study. This took into account advice from the study’s statistician (RH). Participants were drawn from Scotland and northern England.

### Inclusion and exclusion criteria

The Inclusion criteria were: 18 years and over; a member of the farming community; and experiencing mental health problems based on the completion over the phone of a PHQ-9 questionnaire and a score of >  = 8 [25, 26].

Exclusion criteria were: those considered at baseline to be at significant risk of suicide based on a phone interview; currently undertaking or having engaged with CBT or other psychotherapy within the past 6 months; unable to communicate in English; or unable to give informed consent.

### Recruitment

Recruitment was conducted via signposting by local Vets, farming social media, farmer support organisations (but excluding RSABI, who were leading on the provision of the alternate intervention, so as not to engender bias in participant preferences), and farming and mental health charities and support organisations. Flyers and posters were also used, for example, for recruitment at farmers marts. Interested participants were contacted by a researcher directly (phone or e-mail), according to their preference, and provided with a study information pack. If individuals consented to participate, an initial telephone screening call was undertaken to assess eligibility and to obtain informed consent for inclusion. Baseline measures were collected (see Table 1) alongside demographic characteristics (age, sex, ethnicity, educational attainment, employment status/job role). The number of data points assessed were kept to a minimum on advice from the focus groups and farming advisory group.

**Table 1:** Study data collection points

	Eligibility	Baseline	3 months	6 months
Demographic characteristics	X			
PHQ-9 and suicidal ideation	X	X	X	X
3 item sense of coherence		X	X	X
EQ-5D		X	X	X
Engagement with the interventions			X	X

### Randomisation

**Fig. 1:**
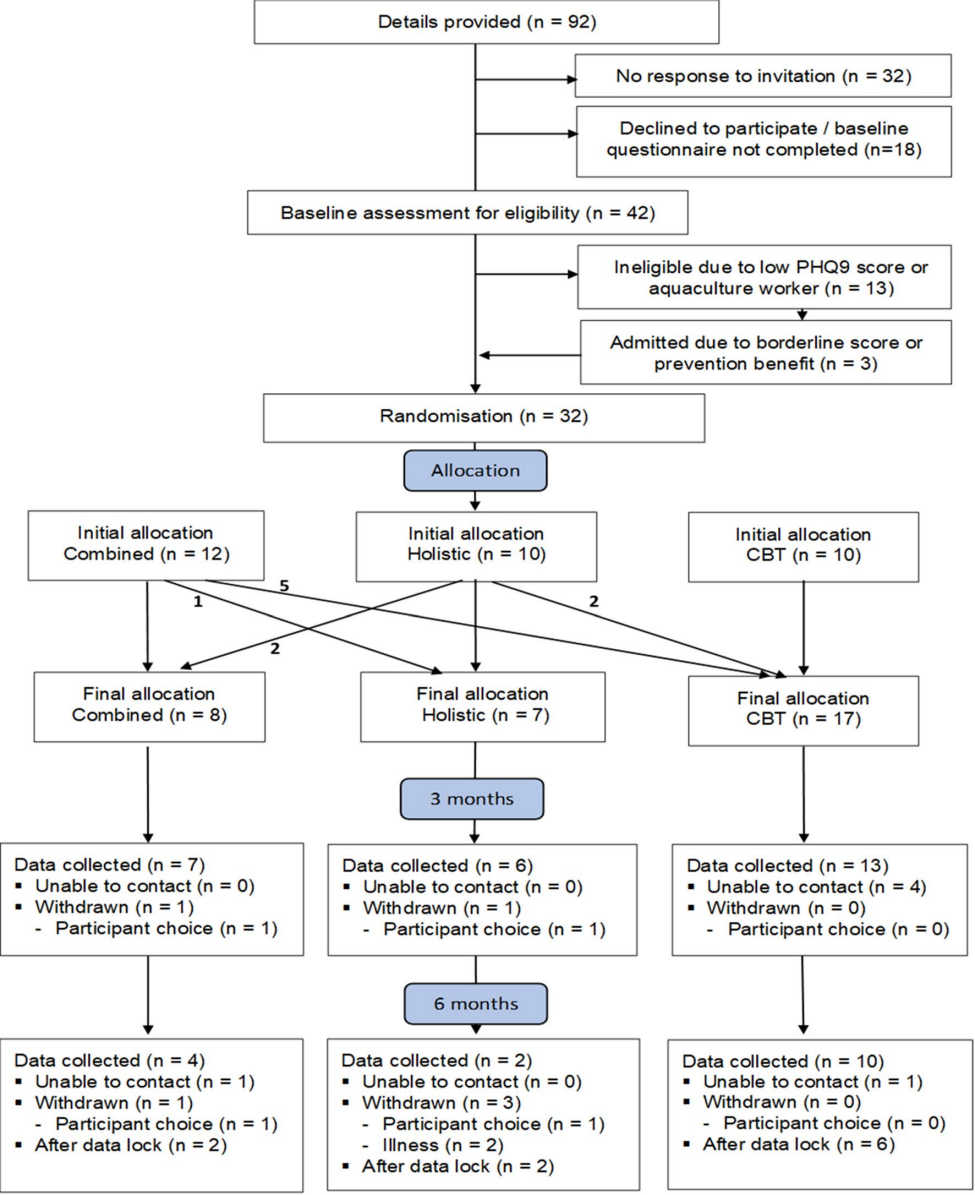
Flow chart of participants

Randomisation was undertaken by full randomisation of the order of subjects, without any blocking, using the rand function within MS Excel. The randomisation options were: (1) to receive the on-line LLTTF-F CBT intervention; or (2) the RSABI social and emotional support intervention); or (3) to receive both interventions combined. Participants were allowed to switch intervention, if they indicated that they had a strong preference to do so before commencement of the intervention to which they had been randomised. All participants were offered this choice. The participants that changed intervention are shown in the study flowchart (Fig. 1). We have undertaken ‘per protocol' analysis rather than intention to treat.

### Living life to the full for farming communities intervention (± telephone support)

This intervention was based on the Living Life to the Full CBT on-line intervention, which was adapted for the farming community. The resource was provided by Five Areas Ltd, which is associated with one of the authors (CW). The programme focuses on educational online life-skills resources for people experiencing stress, anxiety and depression. It was available to participants as an online resource or as a booklet. The intervention used accessible language and included self-guided behavioural change tools.

Participants worked at their own pace but were encouraged to complete the intervention within eight weeks. The on-line version tracked module participation and completion, and additional data such as pages accessed, frequency of access, and for how long etc.

LLTTF participants were given the option of telephone support, consisting of an initial 30-min session, followed by three additional support sessions. Telephone support sessions followed a standard support algorithm and were provided by trained and supervised coaches.

### The social and emotional support intervention

RSABI had an established intervention, which supported people from the Scottish agriculture community emotionally, practically and financially in times of need, providing a holistic service for individuals from the farming community to help them move forward in difficult times, for example, by supporting business reviews, providing access to counselling and providing help with essential living expenses. RSABI offered a home visit service, and a helpline staffed by mental health first aid trained staff and volunteers from 7 am to 11 pm, 365 days of the year, and a ‘call out’ service. Those receiving the RSABI holistic social and emotional support service, had the method of both initial and further contact recorded (telephone, on-line, home visit), along with the number of contacts, and, the types of help accessed (e.g., financial), although information on the results of this contact is not reported in detail in this paper.

### Consent

To ensure support for vulnerable participants, we obtained prior consent from all participants to contact their GP if participants were considered at risk of suicide or showed evidence of deteriorating mental health as identified by their PHQ-9 scores. All participants were also made aware of helplines (such as the Samaritans and Breathing Space).

### Duration of follow up

Baseline measurements were undertaken by telephone (or face to face if this was requested and a visit was feasible), as were follow up measurements at 3 months, and where possible at 6 months. A proportion of participants could not be followed up at 6 months because the study ran out of time before those who had been recruited late in the project reached their 6 month milestone. This was in large measure due to the impact of the COVID-19 pandemic.

### Outcome measures

The pre-agreed primary outcome for the RCT was the change in the measure of depression and anxiety assessed by a difference of mean scores within groups and between groups. Secondary outcomes, related to Sense of Coherence, and EQ-5D, will be reported elsewhere.

Process evaluation outcomes were: recruitment, retention, and engagement with the different interventions. Three engagement questions were asked: *Q1. Did you find the intervention you received *via* this study helpful?* (Not at all useful, Of small benefit, Not sure, Quite helpful, Very helpful). *Q2. Did you follow the course/advice as instructed?* (No I did not, To a small degree, To a reasonable degree/mostly, Always). *Q3. Would you say you completed the course/sessions that were offered?* (Not at all/not much, Only partly/to a small degree, Mostly, Yes as instructed/offered).

### Ethical approval

Ethical approval was granted by Stirling University General University Ethics Panel Reference: GUEP (19 20) 901. This study was performed in line with the principles of the Declaration of Helsinki. Informed consent was obtained from all participants. The intervention is reported in accordance with CONSORT guidelines (See Supplementary Material for assessment of the study against the guideline’s criteria).

## Results

### Changes to PHQ-9 scores

All three interventions showed a positive change in PHQ-9 scores with the LLTTF-F CBT-based intervention showing most improvement as follows: Combined intervention mean baseline score was 18.1 compared to 12.0 at 3-month follow-up (mean change 6.1); Telephone accessed social and emotional support mean baseline score was 11.3 compared to 6.7 at 3-month follow-up (mean change 4.6); CBT-based intervention only mean baseline score was 11.8 compared to 4.5 at 3-month follow-up (mean change 7.3).

A summary of baseline demographic features is provided in Table 2. Ethnicity is not shown in the table as all participants were White Scottish or British.

**Table 2:** Demographic characteristics by intervention (after personal preferences were incorporated)

Intervention category	Number of participants	Mean age (yrs)	Sex		What proportion of your income is derived from farming?			Which of these options best describes your formal education?		
			Male	Female	> 10%	10–50%	> 50%	Primary/secondary school	Secondary advanced /vocational / further education	University/postgraduate /professional
LLTTF-F CBT	8	51.6	6 (75%)	2 (25%)	2 (25%)	1 (12.5%)	5 (62.5%)	1 (12.5%)	5 (62.5%)	2 (25%)
Social and emotional support	7	53.6	6 (100%)	0 (0%)	0 (0%)	1 (17%)	5 (83%)	3 (43%)	2 (28.5%)	2 (28.5%)
Both interventions	17	52.2	13 (76.5%)	4 (23.5%)	4 (22%)	1 (5.5%)	13 (72.5%)	1 (6%)	7 (41%)	9 (53%)

### Recruitment

We recruited and subsequently randomised 32 out of our target of 40 participants (80% of target). Face to face recruitment by someone with recognised farming or rural links was considered as the most successful mechanism. Engaging farmers in research was possible but required significant, focused effort.

Twenty nine of the 42 participants screened for eligibility meet our initial inclusion criteria (eligibility of 69%). Based on researcher discussion, three participants initially classed as ‘ineligible’ were subsequently included as ‘borderline eligible’ and included in the study. Allocation details are shown in Fig. 1.

### Retention

Retention rates were good, as 26 of the 32 participants (81%) provided follow-up data at three months, and 16 out of the 22 participants who were contacted at six months (73%). The main reason that only 22 out of 32 participants completed six-month questionnaires was that ten participants (31%) had been participating in the study for less than six months when the study ended, as the project data collection stage had run out of time. The late recruitment of these 10 participants to the study was related to project delays due to COVID-19 pandemic restrictions that were nationally applied to research activity.

### Allocation

Initial random allocation was fairly evenly distributed: emotional/social support (n = 10); on-line/booklet LLTTF-F CBT ± telephone support (n = 10); and combined (n = 12). However, allowing for a preference to be expressed resulted in final allocations of: emotional/social support (n = 7); on-line/booklet LLTTF-F ± telephone support (n = 17); and combined intervention (n = 8). In summary, more farmers indicated a preference to switch to the LLTTF-F CBT-based intervention than the emotional/social support intervention, or both interventions combined.

### Engagement

Assessment of engagement indicated that all the interventions were acceptable, but suggested that engagement could be enhanced further in future studies. Maximal engagement with the support materials was primarily prevented by participant’s perceived ‘lack of time’, with service users stating that this was ‘too much on top of everything else’ in their lives. Guided, personal telephone support was very valued, especially in the early phase of engagement with the support material.

### Engagement with the interventions

Of those receiving the combined intervention: 3/5 reported following advice/course to a small degree, and 2/5 mostly/always followed advice/course. All completed the course at least ‘to a small degree’. On-line usage data showed that an average of nine modules were accessed.

Of those receiving telephone accessed social and emotional support, 4/5 reported following advice/course mostly as instructed, and 1/5 to ‘a small degree’. In answer to the question “Would you say you completed the course/sessions that were offered?” 2/5 ‘mostly’ completed the sessions that were offered, 2/5 to ‘a small degree’ and 1/5 ‘not at all/not much’. The mean time provided by the telephone accessed social and emotional support service was 4 h 17 min (median 2 h 15 min) and the average number of contacts overall was 29 contacts per participant (median 23). The majority (4/6 responders) felt they had followed the advice received.

Of those receiving the LLTTF-F—intervention only, 3/13 reported following advice/course to a small degree, 5/13 mostly/always followed advice/course, and 5 reported they did not follow advice/course. Four completed the course ‘to a small degree’, 5 completed ‘mostly as instructed’ and 4 followed the course ‘not at all/not much’. On-line usage data from 9/13 participants showed an average of 6 modules accessed.

### Effect of telephone support

More positive support was expressed for the LLTTF-F CBT based intervention when it was accompanied with telephone support, as 23/25 (92%) of those who received the LLTTF-F intervention opted for telephone support. In addition, 5/25 (25%) requested booklets to supplement, or to provide an alternative, to the online LLTTF-F.

### Summary of process evaluation

A key finding is that the two interventions appeared to meet different needs. The CBT intervention had the largest effect on PQ-9 scores (in this small sample), whilst the practical support offered within the holistic social support intervention was particularly valued for other aspects, such as helping to make difficult business decisions.

## Discussion

All the interventions resulted in reductions in PHQ-9 scores, providing some indication that the various components of the two interventions were of benefit. This was particularly the case for the CBT intervention, where there was a mean fall in PHQ-9 scores of 7.3 (from 111.8 to 4.5). This suggests that CBT may be best at addressing immediate the specific mental health issues. However, such an approach would miss out on aspects of the social support that were very highly valued, and which it is possible to speculate may help to prevent later mental health problems.

Perhaps an optimised intervention for the farming community, based on the study data on engagement, and published elsewhere [17], would be a combination of supported (and possible face-to-face) start-up session(s) alongside a CBT-based intervention. There was limited engagement with written material by some participants, and it is possible to speculate that supplementary video and audio content (such as podcasts) might be helpful, and might reflect farmers lifestyle/ Listening to material whilst working might address a perceived ‘lack of time’. Practical support for farm related problems is valued but resource intensive.

### Strengths and weaknesses

A number of lessons were learned from process measures to inform a larger RCT. We recruited > 70% of target (80% achieved, or 73% when removing the three who were initially ‘borderline eligible’ who were still entered into the study. However, recruitment was labour intensive. We met our target of ≥ 40% of contacts being eligible (62% eligible), and our target of < 30% attrition (only 4 participants withdrew (12.5%), with a further 2 (6%) classed as ‘unable to contact’), leading to overall attrition of 16% at 3 months and 27% at 6 months.

The sample size was small, and we have therefore not undertaken statistical analysis beyond the difference in baseline and three-month PHQ-9 scores. Given the sample size, predominant male gender and white Scottish or British ethnicity, caution should be applied in terms of generalisability to other settings. For logistical and ethical reasons, it was not possible to include a control and this is a weakness that should be considered in further studies.

We recognise that a feasibility study does not require a power calculation, and that our approach to sample size is open to critique. However, given the emphasis on the CONSORT checklist on a power calculation, this was undertaken.

The study would have been strengthened by using a formal structure to assess the components of the low intensity support that was provided by RSABI volunteers, in line with the taxonomy developed by Glasgow and Rosen[27].

There was significant variation in baseline PHQ-9 baseline scores. This may simply be as a result of the small sample size, but it is possible to speculate that it may also indicate some self-selection by participants with different types of needs. The PHQ-9 tool has the advantage of being shorter than HADS, which was initially considered as a measure of mental health. Discussions with our project reference group (including farmers and farmer representatives) concluded that the PHQ-9 was considered to be more acceptable to individuals in the farming and crofting community, in terms of its comprehension and wording, and therefore also for telephone completion. The PHQ-9 does include a question on fatigue, and as farmers work very long hours, this question may have picked up fatigue that was not associated with mental health.

The approach that we took did not include a control group. A waiting list control design would have been an alternative approach, which would have provided a control arm to the study. However, this study was a feasibility study which also focused on establishing ‘preferences’ and therefore studying preferences and engagement with the interventions was prioritised.

### Comparison with other literature

Interactive computerised cognitive behaviour therapy has been found to be acceptable in U.S. rural communities in relation to privacy, accessibility, user-friendliness and cultural appropriateness [13]. However, there is little current knowledge concerning preferences and acceptability or up-take of remote interventions in a UK context, and how these can best be signposted to farmers. This study indicates that people in the farming community in Scotland experiencing mental health issues or personal stresses may be willing to engage with online material, but there is still a significant appetite for supplementary paper-based resources.

There has been a recent encouraging growth in studies addressing mental health and suicide prevention in farming communities, with the publication of a range of qualitative studies in different parts of the world [14, 28–31], and a number of intervention studies [32–34]. These studies suggest the benefits of multi-modal approaches that are tailored to the farming community, as the aetiology of problems in this community is diverse. Supportive interventions clearly need to be incorporated into national strategy on farming and agricultural community development [35–37].

## Conclusion

This study has provided evidence that those from the farming community can be recruited, although recruitment requires intense effort, and can be retained in an RCT for at least three months. There is evidence that the interventions used were feasible, and tentative evidence that these had a demonstrable benefit to mental wellbeing, using standard available measurement tools.

### Supplementary Information

Below is the link to the electronic supplementary material.

Supplementary file1 (DOC 108 KB)

## Data Availability

The datasets generated and/or analysed during the current study are available from the authors on reasonable request.

## Abbreviations

LLTTF-F: Living Life to the Full for Farming Communities
RSABI: Royal Scottish Agricultural Benevolent Institution
